# Aptamer-Based Imaging of Polyisoprenoids in the Malaria Parasite

**DOI:** 10.3390/molecules29010178

**Published:** 2023-12-28

**Authors:** Flavia M. Zimbres, Emilio F. Merino, Grant J. Butschek, Joshua H. Butler, Frédéric Ducongé, Maria B. Cassera

**Affiliations:** 1 Department of Biochemistry and Molecular Biology, University of Georgia, Athens, GA 30602, USA; 2 Center for Tropical and Emerging Global Diseases (CTEGD), University of Georgia, Athens, GA 30602, USA; 3 French Atomic Energy Commission (CEA), Fundamental Research Division (DRF), Institute of Biology François Jacob (Jacob), Molecular Imaging Research Center, 92265 Fontenay-aux-Roses, France; 4 Neurodegenerative Diseases Laboratory, CNRS CEA UMR 9199, 92265 Fontenay-aux-Roses, France; 5 Paris-Saclay University, 92265 Fontenay-aux-Roses, France

**Keywords:** *Plasmodium*, polyisoprenoids, aptamers, aptamer-based imaging

## Abstract

Dolichols are isoprenoid end-products of the mevalonate and 2*C*-methyl-D-erythritol-4-phosphate pathways. The synthesis of dolichols is initiated with the addition of several molecules of isopentenyl diphosphate to farnesyl diphosphate. This reaction is catalyzed by a *cis*-prenyltransferase and leads to the formation of polyprenyl diphosphate. Subsequent steps involve the dephosphorylation and reduction of the α-isoprene unit by a polyprenol reductase, resulting in the generation of dolichol. The size of the dolichol varies, depending on the number of isoprene units incorporated. In eukaryotes, dolichols are synthesized as a mixture of four or more different lengths. Their biosynthesis is predicted to occur in the endoplasmic reticulum, where dolichols play an essential role in protein glycosylation. In this study, we have developed a selection of aptamers targeting dolichols and enhanced their specificity by incorporating fatty acids for negative selection. One aptamer showed high enrichment and specificity for linear polyisoprenoids containing at least one oxygen atom, such as an alcohol or aldehyde, in the α-isoprene unit. The selected aptamer proved to be a valuable tool for the subcellular localization of polyisoprenoids in the malaria parasite. To the best of our knowledge, this is the first time that polyisoprenoids have been localized within a cell using aptamer-based imaging techniques.

## 1. Introduction

The subcellular localization of metabolites is a fundamental aspect of cellular biology, as it provides critical insights into the spatial distribution and compartmentalization of key molecules within the cells. Traditional methods for studying the subcellular localization of metabolites, such as immunofluorescence and chemical tagging, often require the generation of specific antibodies or chemical modifications, which can be time-consuming, labor-intensive, and may suffer from limitations in regards to specificity and sensitivity.

Over the past several years, aptamers have emerged as powerful tools for use in subcellular localization studies [1]. Aptamers are short, single-stranded nucleic acids that can be selected in vitro to bind to a wide range of target molecules, including metabolites [2,3,4], with high affinity and specificity. The unique properties of aptamers, such as their small size, robustness, and ease of synthesis, make them ideal candidates for use in studying metabolite localization. By conjugating aptamers with fluorophores or other imaging tags, it becomes possible to visualize and track metabolites within living or fixed cells, with a spatial resolution that enables the investigation of organelle-specific localization and changes in metabolite distribution to aid in the understanding of cellular metabolism.

Polyisoprenoids are a diverse class of compounds built from isoprene units, a five-carbon molecule with a branched structure, and which can be found varying in size and structure, including linear or branched chains and cyclic structures. Polyisoprenoids play vital roles in many biological processes, including membrane structure, protein modification, and signaling pathways. Linear polyisoprenoids (polyprenols, dolichols, and their phosphate esters and carboxylic acid derivatives) are present in all membrane systems [5,6].

Among their biological functions are the regulation of the membrane fluidity [7,8], the stimulation of spore wall formation in yeast [9], the scavenging of free radicals in cell membranes [10,11,12], and a crucial role in the modification and biosynthesis of glycoproteins, glycolipids, and glycosylphosphatidylinositol (GPI)-anchored proteins. Thus, understanding the subcellular localization of polyisoprenoids is critical, as it provides insights into their spatial distribution and their potential involvement in cellular functions.

The subcellular localization of polyisoprenoids, such as dolichols, in the malaria parasite is of great importance due to their participation in vital biological processes [13]. Malaria, caused by *Plasmodium* parasites, remains a major global health concern, and the search for new therapeutic targets remains a high priority for overcoming the problem of antimalarial drug resistance [14]. Polyisoprenoids have been implicated in essential pathways within the malaria parasite, which includes serving as a lipid oligosaccharide carrier for protein *N*-glycosylation, *C*- and *O*-mannosylation, and GPI synthesis (reviewed in [15]).

Recently, we reexamined polyisoprenoid biosynthesis in *Plasmodium falciparum* using metabolomics and molecular approaches and revealed an unusual co-occurrence of polyprenols and dolichols [16]. The co-occurrence of polyprenols and dolichols, i.e., the detection of a dolichol along with significant levels of its precursor polyprenol, is unusual in eukaryotic cells. Interestingly, we also uncovered a distinctive temporal profile of these lipids in the asexual intraerythrocytic developmental cycle of *P. falciparum*. Therefore, investigating the subcellular distribution and localization of polyisoprenoids in the malaria parasite can provide valuable insights into the metabolism and biology of the parasite, potentially identifying new targets for antimalarial interventions.

Despite the biological significance of polyisoprenoid in the malaria parasite, studying their subcellular localization presents significant challenges. Primarily, difficulties in the chemical synthesis of polyisoprenoids have limited the availability of fluorescently tagged analogs required for their precise subcellular localization. To our knowledge, the identification of aptamers that recognize lipids lacking large polar modifications, such as dolichol and polyprenol, by conventional systematic evolution of ligands by exponential enrichment (SELEX) screening has not yet been reported. The major challenge is that polyisoprenoids are soluble only in organic solvents, such as hexane, in which aptamers will precipitate.

In this study, we were able to overcome this limitation by developing a modified SELEX technique in which lipids are layered on a glass surface. The resulting aptamer, designated as Apt^PP^, exhibited affinity, not only for dolichol, but also for other polyisoprenoids containing an oxygen atom in the form of alcohol or aldehyde in the α-isoprene unit. Moreover, aptamer-based cell imaging revealed a distinctive subcellular localization pattern of Apt^PP^ at different stages of the malaria parasite’s life cycle, as well as in response to both the chemical and genetic modulation of isoprenoid biosynthesis, highlighting its potential as a promising metabolite detection approach in this parasite.

## 2. Results and Discussion

### 2.1. Selection of Aptamers against Polyisoprenoids

Compared to non-membrane molecular targets, the successful selection of aptamers for membrane molecular targets has been relatively limited [17]. Notably, in the case of membrane proteins, various selection methods have been employed, including the use of soluble protein fragments, detergent–membrane protein mixed micelles, whole cells, vesicles derived from cellular membranes, and enveloped viruses [18]. For targeting membrane lipids, liposomes have served as an experimental system for aptamer selection [17].

**Figure 1:**
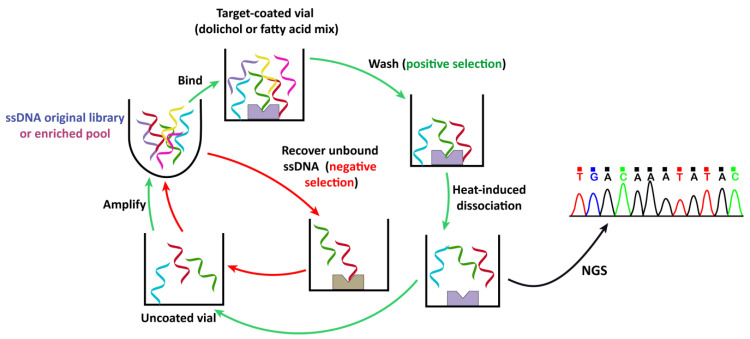
Schemes of the positive and negative selection cycles are illustrated. During positive selection, the desired ssDNA sequences were enriched through binding to the dolichol mixture, while during negative selection, unwanted sequences were removed by their successive interactions with monounsaturated and polyunsaturated fatty acids coated on a glass surface. For positive selection, the folded ssDNA pool was incubated with a glass vial coated with a mixture of dolichols, and unbound ssDNA were recovered for enrichment analysis. The vial containing the dolichol-bound ssDNA pool complex was washed. Then, selection buffer was added and heated to release the bound ssDNA. The recovered ssDNA pool was incubated in an empty (uncoated) glass vial. The supernatant was recovered and precipitated. The enriched sequences were amplified by PCR for the next cycle of selection. For negative selection, a similar procedure was applied, first using a glass vial coated with a mixture of monounsaturated fatty acids. The unbound ssDNA was then recovered and incubated in a vial coated with polyunsaturated fatty acids, followed by selection against an uncoated glass vial. NGS: next-generation sequencing.

In our current study, we aimed to explore a simplified approach for the development of an aptamer capable of specifically recognizing polyisoprenoids while excluding fatty acids (FA) present in common membrane lipids. For this purpose, ten cycles of aptamer selection were conducted using lipids coated on a glass surface, alternating positive and negative selection rounds (Figure 1 and Table 1), with increasing stringency implemented throughout the process (Table 1). The positive selection rounds involved changing the concentration of the single-stranded DNA (ssDNA) pool every three cycles and the dolichol mixture of interest every two cycles, while maintaining a constant concentration of FA mixtures during the negative selection rounds. Two consecutive selections using FA mixtures were employed to prevent non-specific interactions of selected aptamers with other membrane lipids due to structural similarities with the hydrocarbon chain of the polyisoprenoids. Washing steps were employed to eliminate low-affinity and unbound ssDNA. The interaction between the aptamer and the metabolite target was facilitated by a prolonged incubation period, followed by heat-induced dissociation and amplification of the recovered bound ssDNA for the subsequent round.

To assess the efficacy of aptamer selection using lipids coated on a glass surface, we used the non-equilibrium capillary electrophoresis of equilibrium mixtures (NECEEM) method [19]. We first assessed the migration time of the folded ssDNA library at varying concentrations (Figure S1). We then analyzed the portion of the folded ssDNA library that did not bind after incubation with a dolichol mixture. All samples exhibited similar migration times between 15 to 19 min (Figures S2–S4). However, the samples which were incubated with the metabolite displayed reduced signals compared to those of the initial ssDNA library prior to incubation. These results indicate that a small portion of DNA remained attached to the immobilized metabolite target, thus confirming that aptamers have been selected through this process.

### 2.2. Families of DNA Aptamers Obtained through the Selection Process

The enrichment of certain sequences after positive (R04, R06, R09) or negative (R05, R10) selection rounds was analyzed using high-throughput sequencing. Approximately 1.5 million reads were analyzed per round. Of the 6.5 million unique sequences in the analysis, 5745 exhibited a frequency in the library greater than 0.001% in at least one round. These sequences were retrieved and grouped into 5438 families. Multiple alignments of the 200 most enriched families revealed that one family (Family 1) was highly enriched, representing 13.9% of the library after R10 (Table 2 and Supplementary Tables S1 and S2). Moreover, it is noteworthy that the frequency of Family 1 increased significantly in the R06 positive round (2.8%) compared to the R05 negative round (0.01%). Similarly, its frequency was twice as high between the R10 positive selection round (13.9%) and the R09 negative round (5.2%). These results suggested a higher binding of the sequence from Family 1 to the dolichol mixture as compared to the fatty acid mixture. The second most enriched family shared some similarity with Family 1, and also exhibited a higher frequency in R10 (0.04%) compared to R09 (0.01%), but its frequency is very low compared to that of Family 1 (Table 2). Therefore, the most abundant sequence, Family 1, was selected for further validation and was renamed Apt^PP^.

**Figure 2:**
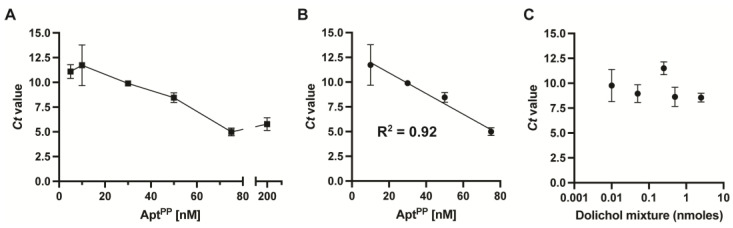
(**A**) The concentration-dependent binding of Apt^PP^ to dolichol was assessed by qRT-PCR analysis performed in triplicate against 2 nmoles of the dolichol mixture. (**B**) The linear regression was calculated between 10 and 75 nM. (**C**) The concentration-dependent binding of 10 nM of Apt^PP^ against varying concentrations of dolichol was determined by qRT-PCR analysis. All experiments were performed in technical duplicate and in at least two independent determinations.

To assess the affinity of Apt^PP^ for dolichols, quantitative real-time PCR (qRT-PCR) was performed, as described previously in Ref. [19]. In this analysis, the *Ct* value is used as a measure of ssDNA bound to a ligand or target molecule, with a lower *Ct* value indicating a higher selectivity for the target molecule. First, we tested varying concentrations of Apt^PP^ against 1 nmole of dolichol mixture (Figure 2A). As expected, the *Ct* value decreased with increasing concentrations of Apt^PP^, supporting a specific and concentration-dependent binding of Apt^PP^ to dolichols (Figure 2B, linear correlation). Then, we varied the concentration of the dolichol mixture. However, no significant changes in the *Ct* values were observed, suggesting that Apt^PP^ has a high affinity for dolichol, with a limit of detection (LOD) < 0.01 nmoles.

### 2.3. Secondary Structure Prediction of Apt^PP^ and Variant Sequence

**Figure 3:**
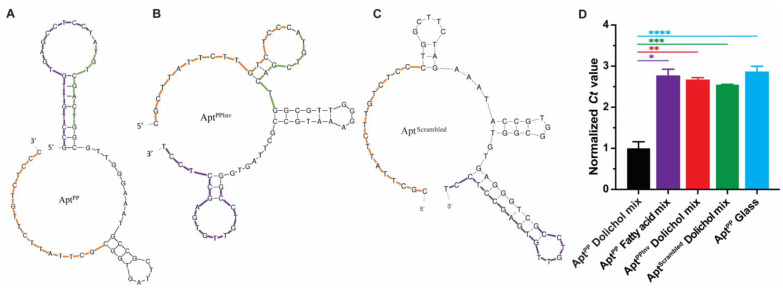
The secondary structures of (**A**) Apt^PP^, (**B**) Apt^PPInv^, and (**C**) Apt^Scrambled^ were predicted using the mFold Web Server. The 5′-constant region is represented in purple, the conserved motif in green, and the 3′-constant region in orange. The constant regions were inverted in the Apt^PPInv^ sequence. (**D**) The affinity of Apt^PP^, Apt^PPInv^, and Apt^Scrambled^ for the dolichol and fatty acid mixture was determined by qRT-PCR analysis, performed in triplicate, using 10 nM of aptamer and 1 nmole of metabolites. Normalized *Ct* values were calculated as a ratio to the *Ct*-dolichol mixture. Low affinity was determined by the *Ct* value obtained for recovered Apt^PP^ after exposure to a glass vial without the metabolite and fatty acid mixture. (*) *p* < 0.0002; (**) *p* < 0.000008; (***) *p* < 0.000001; (****) *p* < 0.00003.

The primary sequence and secondary structure of an aptamer provide insights into potential sites of interaction with the target molecule. The secondary structure consists of nucleotide pairs (stacking pairs) and unpaired bases (loops), with the latter serving as potential interaction sites for other molecules [20]. It is thus expected that conserved motifs within an aptamer are typically located within the loop regions [21]. In the case of Apt^PP^, the secondary structure prediction revealed that the first four nucleotides of the conserved motif (**ATGT**CGACTG) are part of a bulge loop (Figure 3A, sequence in green).

To confirm the functional significance of the conserved motif in Apt^PP^, we generated a variant, while maintaining the overall base composition, as well as a scrambled DNA sequence (Table 3). In the variant, called Apt^PPInv^, the positions of the 5′ and 3′ constant regions were swapped, resulting in structural changes (Figure 3B) and a loss of affinity for dolichols (Figure 3D). A similar result was observed with a scrambled version of the sequence between the constant regions of Apt^PPInv^ (Figure 3C,D). These results indicate that the position of the constant regions, along with the conserved motif, gives rise to a unique structure that can interact with dolichols, thus supporting aptamer specificity.

### 2.4. Specificity of Apt^PP^ for Different Isoprenoid Products

**Figure 4:**
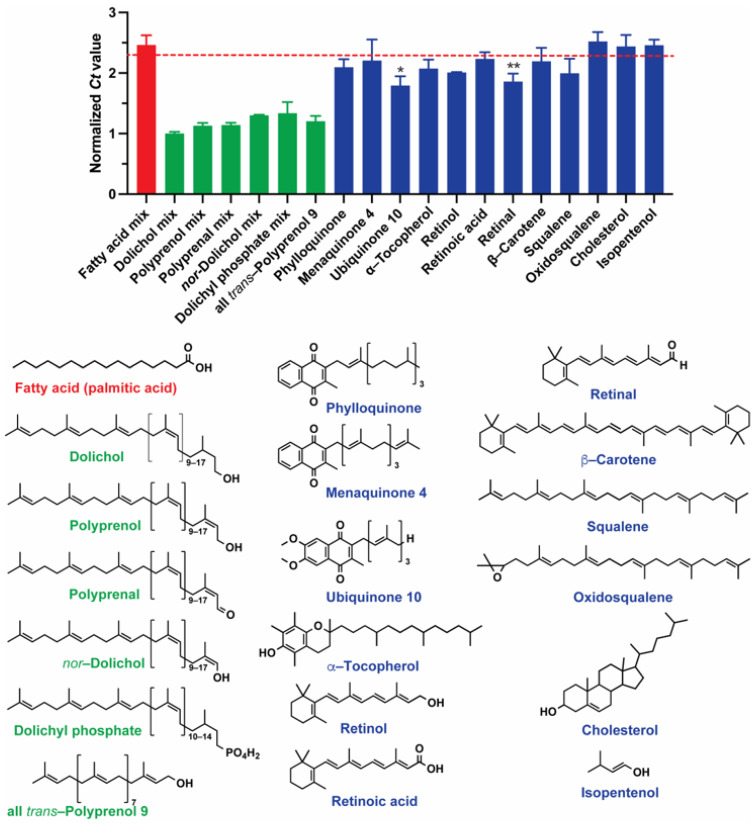
The structure–affinity relationship of Apt^PP^ was assessed by qRT-PCR, in which the lowest *Ct* value indicates higher amounts of bound DNA (higher selectivity). Normalized *Ct* values were calculated as a ratio to the *Ct*-dolichol mixture. The metabolites (1.8 nmoles) were incubated with 12.5 nM Apt^PP^ at room temperature for 1 h. Unbound Apt^PP^ was removed, the metabolites were washed three times, and the bound Apt^PP^ was recovered for the qRT-PCR assays. The values represent the average from three independent assays. The red dotted line indicates a low affinity, as determined by recovered Apt^PP^ after exposure to a glass vial without the metabolite. (*) *p* < 0.005; (**) *p* < 0.003. The structures of each metabolite included in this study are displayed. Palmitic acid is shown as a representative fatty acid (see Section 3.1 for a detailed composition of the mixture). Brackets indicate the range of isoprene units present in the mixture.

Dolichol is a polyisoprenoid alcohol lipid that is composed of repeating isoprene units linked together head-to-tail, and the length of these lipids is variable (Figure 4). The terminal hydroxyl group of dolichols may exist as either free, phosphorylated, or esterified with fatty acids. Polyprenol is the metabolic precursor of dolichol, and the α-terminal isoprene unit is unsaturated (Figure 4).

To investigate the structure–affinity relationship of Apt^PP^ with non-polar polyprenoids in vitro, we conducted qRT-PCR analysis, as previously described by Liao et al. [22]. Apt^PP^ exhibits the specific recognition of linear *cis*- and *trans*-polyisoprenoids that contain at least one oxygen atom in the α-isoprene unit, in the form of alcohol or aldehyde (polyprenal), but not epoxide (2,3-oxidosqualene), as illustrated by the distinct responses observed in Figure 4 (green bars versus blue bars). Moreover, Apt^PP^ showed a higher affinity for dolichol than for dolichyl phosphate (Dol-P) and *nor*-dolichol, a semi-synthetic derivative of dolichol lacking a CH_2_ in the α-isoprene unit. Apt^PP^ did not recognize isopentenol (Figure 4). These findings from our structure–affinity relationship analysis suggest that Apt^PP^ binds to both the α-isoprene unit and the polyisoprenoid chain simultaneously, which contributes to its specificity. In addition, the qRT-PCR analysis to survey the target specificity using lipids layered on a glass surface resulted in a simple approach for structure–affinity relationships that can be expanded to other lipids with diverse chemical structures.

### 2.5. Subcellular Localization of Apt^PP^ in Plasmodium falciparum

**Figure 5:**
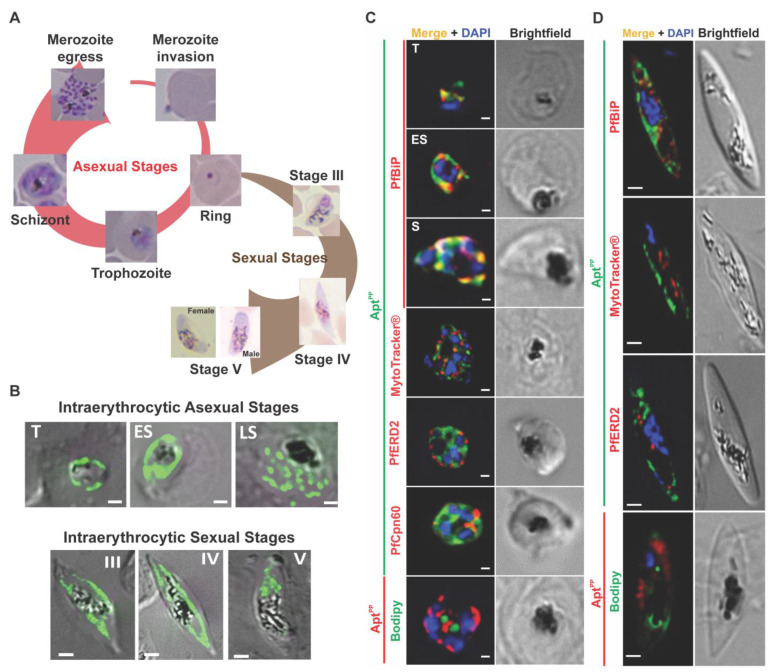
(**A**) Scheme of the intraerythrocytic asexual and sexual stages of *P. falciparum*. (**B**) 6-FAM-Apt^PP^ subcellular localization in the trophozoite (T), early schizont (ES), late schizont (LS), and stage III, IV and V gametocytes. (**C**) Colocalization (orange/yellow) of either 6-FAM-Apt^PP^ (green) or Cy5-Apt^PP^ (red) was assessed by co-staining with MitoTracker (mitochondria, red), PfERD2 (Golgi apparatus, red), PfCpn60 (apicoplast, red) or BODIPY (lipid droplets, green) in the *P. falciparum* schizont stage. The endoplasmic reticulum marker PfBiP (red) was assessed in the trophozoite (T), early schizont (ES), and schizont (S) stages. (**D**) Colocalization (orange/yellow) of either 6-FAM-Apt^PP^ or Cy5-Apt^PP^ was assessed by co-staining with the PfBiP, MitoTracker, PfERD2, and BODIPY markers in the gametocytes (intraerythrocytic sexual stages). Scale bar, 2 µm. DAPI (blue) was used for nuclear staining.

Malaria, caused by *Plasmodium* parasites, remains a major global health concern, and the search for new therapeutic targets is of great importance. Thus, the temporal and spatial visualization of polyisoprenoids in the cellular context of the malaria parasite is important for studying their biological functions and potentially developing novel strategies to combat the disease [15,23,24]. The life cycle of *P. falciparum* begins with the transmission of the parasite to humans through the bite of an infected female *Anopheles* mosquito. The parasite then undergoes a sequence of developmental stages, beginning with the invasion of *P. falciparum* sporozoites into liver cells, which then develop into merozoites, which are released into the bloodstream. The merozoites invade the red blood cells to start the asexual intraerythrocytic developmental cycle (Figure 5A) responsible for the clinical manifestation of malaria. Following invasion, the merozoites progress sequentially through the ring and trophozoite stages, followed by schizogony that forms new merozoites which will exit the host cell to infect fresh red blood cells, thus beginning a new intraerythrocytic developmental cycle. During this intraerythrocytic stage, some parasites differentiate into sexual-stage forms called gametocytes. *P. falciparum* gametocytes develop through five morphologically distinct stages (I to V), requiring 10 to 12 days to fully mature into stage V gametocytes.

In our previous study [16], we conducted untargeted lipidomic analyses using a liquid chromatography—high-resolution mass spectrometry (LC-HRMS) system. Our focus was on the asexual and sexual intraerythrocytic developmental cycle of *P. falciparum*, for which we identified distinct temporal profiles of both polyprenols and dolichols. As mentioned above, the detection of a dolichol, along with significant levels of its precursor polyprenol, is unusual in eukaryotic cells. Notably, we found that the dolichol/polyprenol ratios changed between the different stages of the parasite’s development. Schizont stages displayed ratios closer to one, while ring and trophozoite stages exhibited significantly higher ratios compared those of the schizonts. An intriguing finding was that in the stage IV gametocytes, dolichols were present, while polyprenols were nearly undetectable. Furthermore, human red blood cells (RBCs) contained low levels of dolichols, as expected. Considering the prevalence of polyisoprenoids in eukaryotic cell membranes and our metabolomics studies, we hypothesized that the distinctive temporal profile of polyprenols and dolichols during the asexual and sexual intraerythrocytic life cycles of the malaria parasite may correlate with different subcellular localizations, implying different biological roles during these developmental cycles. This hypothesis served as the driving force behind the development of an aptamer suitable for aptamer-based fluorescence microscopy, which aimed to complement our previous metabolomics and molecular studies [16].

To evaluate the effectiveness of Apt^PP^ for aptamer-based fluorescence microscopy, we obtained a commercially synthesized Apt^PP^, modified with either 5′-6-FAM (designated as 6-FAM-Apt^PP^, emitting green fluorescence) or 5′-Cy5 (designated as Cy5-Apt^PP^, emitting red fluorescence). The fluorescently labeled Apt^PP^ were then incubated with fixed samples of different intraerythrocytic stages of *P. falciparum*, following the procedure outlined in the Methodology section. Remarkably, our experiments revealed a distinct labeling pattern of Apt^PP^ throughout both the asexual and sexual intraerythrocytic life cycle of the malaria parasite (Figure 5B). This observation suggests that polyisoprenoids exhibit specific subcellular localizations that undergo changes during the life cycle of the parasite. These results serve as compelling evidence that our Apt^PP^ is well-suited for in situ aptamer-based metabolite imaging. Importantly, to the best of our knowledge, this is the first time polyisoprenoids have been successfully localized within a cell using this technique.

To further investigate the extensive localization patterns observed, we conducted co-staining experiments using 6-FAM-Apt^PP^ or Cy5-Apt^PP^, along with a panel of markers targeting specific subcellular compartments. These markers included anti-PfBiP for the endoplasmic reticulum, anti-PfERD2 for the Golgi apparatus, anti-Cpn60 for the apicoplast, MitoTracker^TM^ for mitochondria, BODIPY 493/503 for the lipid droplets, and DAPI for the nuclei. Our results showed the robust colocalization of Apt^PP^ with PfBiP in the endoplasmic reticulum during the asexual stages (Figure 5C, Pearson’s coefficient = 0.75). This colocalization was not exclusive to the endoplasmic reticulum organelle, suggesting that polyisoprenoids are also present in other subcellular localizations. We also observed weak colocalization of Apt^PP^ with anti-Cpn60 (Pearson’s coefficient = 0.45), a marker for the non-photosynthetic plastid, called the apicoplast, that shares homology with chloroplasts found in algae and plants. Recent studies have demonstrated active polyprenol synthesis in the apicoplast of the malaria parasite, but the biological function in this organelle remains to be elucidated [24]. Similarly, weak colocalization was observed in the mitochondria, where ubiquinones are synthesized (Pearson’s coefficient = 0.36). Although ubiquinones are not specifically recognized by Apt^PP^, it does recognize *trans*-polyprenol 9 (Figure 4), which is a precursor of ubiquinone in the malaria parasite [25,26]. This suggests a potential recognition of polyprenyl diphosphates by Apt^PP^, similar to that of dolichyl phosphate (Figure 4). In mammalian cells, dolichol has been reported to localize in the Golgi apparatus [27,28]. However, *P. falciparum* possesses a more rudimentary Golgi apparatus, consisting of dispersed and unstacked *cis*- and *trans*-cisternae, which may explain the observed weak colocalization (Pearson’s coefficient = 0.41) [29,30]. No colocalization was observed in the nuclei and lipid droplets. Interestingly, polyprenols were detected in the lipid droplets of sporulating yeast [9] and in the neuromelanin organelles in the brain [31]. Additionally, some areas labeled by Apt^PP^ did not exhibit colocalization with any of the markers used in the asexual stages. Surprisingly, a weak colocalization was observed only with PfBiP in the gametocytes (Pearson’s coefficient = 0.32), indicating a distinct subcellular localization of polyisoprenoids in these stages of the parasite’s intraerythrocytic developmental cycle (Figure 5D). These findings warrant further investigation to determine the composition and biological functions of polyisoprenoids in these specific subcellular compartments.

### 2.6. Validation of the Specificity of Apt^PP^ within the Cellular Environment

**Figure 6:**
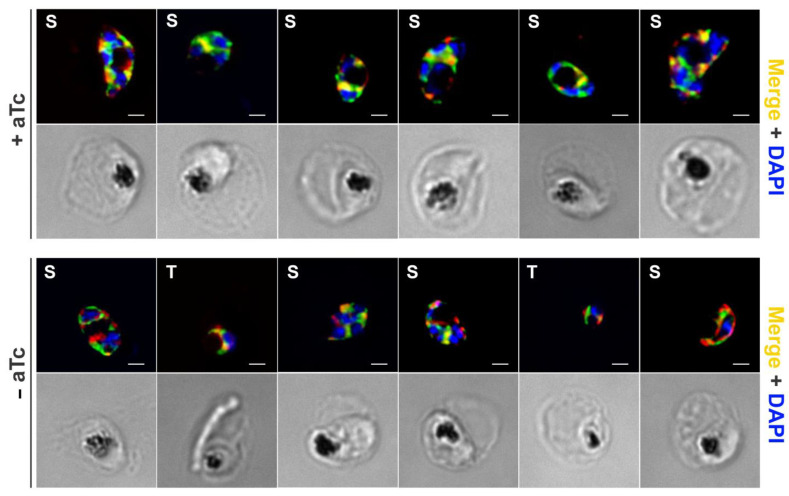
Apt^PP^ subcellular localization in the trophozoite (T) and schizont (S) stages of the intraerythrocytic asexual cycle under normal PfPPRD expression (+aTc) or after the PfPPRD knockdown (-aTc) was induced over 8 days. Colocalization (orange/yellow) of 6-FAM-Apt^PP^ (green) was assessed by co-staining with the PfBiP (red). Samples were collected from three independent experiments. Scale bar, 2 µm. DAPI (blue) was used for nuclear staining.

To validate the specificity of Apt^PP^ within the cellular environment, we assessed its response to the genetic modulation of isoprenoid biosynthesis. Recently, we published findings on the inducible knockdown of polyprenol reductase (PfPPRD), an enzyme responsible for converting polyprenol into dolichol in the malaria parasite [16]. The PfPPRD knockdown was induced by removing anhydrotetracycline (aTc), resulting in the accumulation of polyprenol and in reduced levels of dolichols in these parasites [16]. During these metabolomics experiments, samples from cultures containing the PfPPRD knockdown system were collected and incubated with Apt^PP^ and PfBiP to investigate any potential changes in the colocalization of polyisoprenoids. In the presence of aTc, the parasites exhibited a strong partial colocalization of Apt^PP^ with PfBiP (Figure 6, Pearson’s coefficient = 0.62), similar to that of the wildtype parasites (Figure 5C). However, in the absence of aTc, which prevents PfPPRD protein expression and leads to alterations in polyprenol and dolichol levels [16], a weak partial colocalization of Apt^PP^ with PfBiP (Pearson’s coefficient = 0.39), or the absence thereof, was observed (Figure 6). These findings indicate a distinct subcellular distribution of these isoprenoids within the malaria parasite. Specifically, our data suggest the presence of dolichols, primarily within the endoplasmic reticulum, as their levels were significantly reduced in the PfPPRD knock-down parasites [16], while polyprenols are present in a different subcellular location.

**Figure 7:**
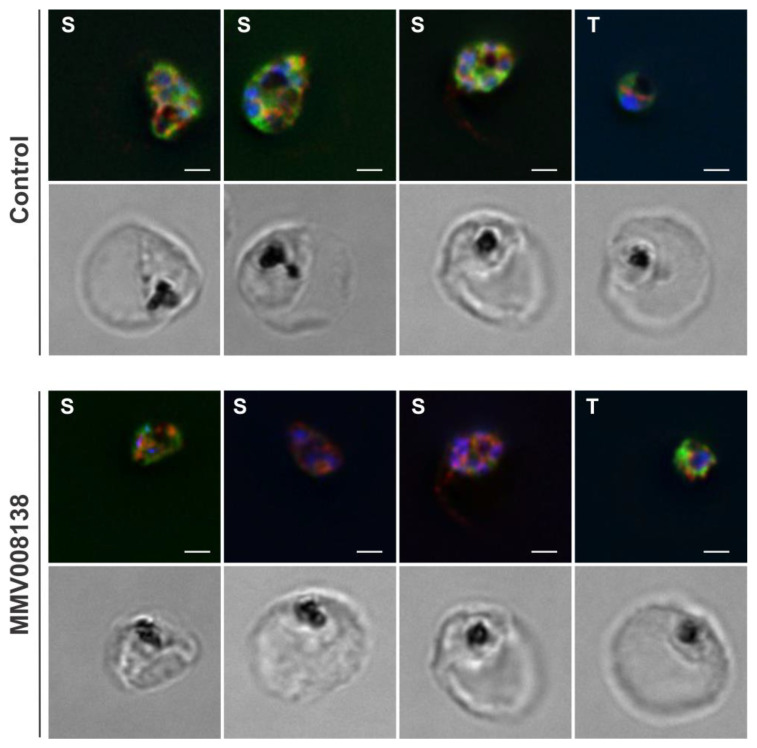
Apt^PP^ subcellular localization in the trophozoite (T) and schizont (S) stages of the intraerythrocytic asexual cycle treated with 1 μM of MMV008138 for 15 h. Colocalization (orange/yellow) of 6-FAM-Apt^PP^ (green) was assessed by co-staining with the PfBiP (red). Samples were collected from two independent experiments. Scale bar, 2 µm. DAPI (blue) was used for nuclear staining.

To further investigate the potential of Apt^PP^ for detecting changes in polyisoprenoid levels within the malaria parasite, we explored the effects of chemical inhibition on isoprenoid product biosynthesis using MMV00813829 [32,33]. This compound specifically targets PfIspD in the 2*C*-methyl-D-erythritol-4-phosphate (MEP) pathway, which is responsible for synthesizing the isoprene unit isopentenyl diphosphate, thus inhibiting the biosynthesis of all isoprenoid products in malaria parasites. To achieve this, synchronous *P. falciparum* cultures in the late ring and early trophozoite stages were treated with 1 μM of MMV008138 for 15 h. As shown in Figure 7, some parasites exhibited reduced Apt^PP^ labeling upon treatment, although not all screened parasites showed the same response, and a strong partial colocalization of Apt^PP^ with PfBiP was still detected (Pearson’s coefficient control = 0.80; Pearson’s coefficient MMV008138 = 0.84). The observed labeling of polyisoprenoids by Apt^PP^ in the treated parasites was anticipated, as the complete depletion of all available isoprenoid products is not achieved following the inhibition of de novo isoprenoid biosynthesis.

Collectively, these results support the specificity of Apt^PP^ in in vivo interactions with polyisoprenoids, allowing us to canvas the dynamic localization of these metabolites in response to genetic or chemical interventions within the malaria parasite. As a result, we have laid the groundwork for further investigations into the biological functions of polyisoprenoids, paving the way for a deeper understanding of their role in malaria parasite biology.

### 2.7. Beyond Polyisoprenoid Detection

Our study has demonstrated the potential of Apt^PP^ as a tool for studying polyisoprenoids in the malaria parasite. The successful optimization of these aptamers for specific targets unlocks exciting opportunities, not only for applications in the malaria parasite but also for exploring their utility with other organisms, such as mammalian cells. We anticipate that our findings will serve as a valuable foundation for researchers to build upon, potentially adapting this SELEX approach for a wide range of bioactive compounds and cellular contexts. By harnessing the power of a simple coated glass and expanding its scope, our hope is that this work will inspire further investigations and innovative discoveries in aptamer-based imaging technologies.

## 3. Materials and Methods

### 3.1. Chemicals

The culture media RPMI 1640, HEPES, gentamycin, and Albumax I were obtained from GIBCO Life Technologies (Thermo Fisher Scientific, Waltham, MA, USA). Glucose, sodium bicarbonate, and hypoxanthine were purchased from Millipore-Sigma (Burlington, MA, USA). Polyprenol, dolichol, polyprenal, and nor-dolichol mixtures of 13 to 21 isoprene units were obtained from Avanti Polar Lipids (Alabaster, AL, USA). The dolichyl phosphate mixture (14 to 18 isoprene units), all trans-polyprenol (9 isoprene units), and isopentenol were obtained from Isoprenoids LC (Tampa, FL, USA). The following chemicals were obtained from Cayman Chemicals (Ann Arbor, MI, USA): polyunsaturated fatty acid mixture (adrenic acid, arachidonic acid, dihomo-γ-linolenic acid, docosahexaenoic acid, docosapentaenoic acid, eicosapentaenoic acid, linoleic acid, α-linolenic acid, and γ-linolenic acid, stearidonic acid), saturated and monounsaturated fatty acid mixture (arachidic acid, lauric acid, lignoceric acid, myristic acid, nervonic acid, oleic acid, palmitic acid, palmitoleic acid, and stearic acid), and ubiquinone-10. Squalene, 2,3-oxidosqualene, cholesterol, β-carotene, menaquinone-4, α-tocopherol, phylloquinone (vitamin K1), retinal, retinoic acid, and retinol were purchase from Millipore-Sigma (St. Louis, MO, USA). Anhydrotetracycline was obtained from Cayman Chemical (Ann Arbor, MI, USA).

### 3.2. Systematic Evolution of Ligands by Exponential Enrichment (SELEX)

The process of selecting DNA aptamers began by amplifying a large-scale random oligonucleotide library. The synthetic library consisted of a random region of 34 nucleotides, surrounded by two constant regions, for primer annealing [34]. The library was amplified by conventional PCR using 1 pmol/µL of modified forward primer (5′-polyA-GCCTGTTGTGAGCCTCCT-3′) and labeled reverse primer (5′-FAM-18C-GGGAGACAAGAATAAGCG-3′), 1× Taq buffer with 20 mM (NH_4_)_2_SO_4_, 50 mM betaine, 5% DMSO, 0.25 mM MgCl_2_, 0.1 mM dNTP mixture, and 5 U of Taq-DNA polymerase in a 100 μL final volume. The amplification products were combined and precipitated with additives consisting of 10% (*v*/*v*) of 3 M sodium acetate (pH 5.2), 10% (*w*/*v*) of 0.1% glycogen, 5% (*v*/*v*) of 1 M MgCl_2_, and completed with three volumes of absolute cold ethanol. After 16 h of incubation at –20 °C, the double-stranded DNA (dsDNA) was pelleted down at 17,000× *g* for 30 min, washed with cold 70% ethanol by tube inversion, and centrifuged at 17,000× *g* for 15 min. The DNA pellet was resuspended in formamide, denatured for 30 min at 95 °C, and loaded into a denaturing polyacrylamide gel (6 M urea) that was pre-run at 400 V for at least 30 min. The reverse single-stranded DNA (5′-FAM-18C ssDNA) was extracted from the gel with phenol:chloroform:isoamyl alcohol (25:24:1, *v*/*v*) (pH 8.0) and precipitated overnight, as described above.

The ssDNA was resuspended in ultrapure water and quantified using a nanodrop. The desired amount of ssDNA was adjusted to a final volume of 100 μL, consisting of 20 µL of selection buffer (SB, 5× stock solution: 125 mM HEPES-NaOH at pH 7.4, 7 mM KCl, 725 mM NaCl, 6 mM MgCl_2_, 9 mM CaCl_2_-2H_2_O and 50 mM D-glucose) and 80 µL H_2_O. The ssDNA pool was denatured at 85 °C for 20 min, followed by refolding at 24 °C.

For a positive selection, folded ssDNA was added to a glass vial coated with the dolichol mixture (see Table 1 and Figure 1) and incubated at room temperature (RT) for 1 h. The supernatant, containing the unbound ssDNA pool, was recovered for sequence analysis. The vial containing bound ssDNA was washed with 1× of SB. To dissociate the ssDNA bound to the metabolite, 200 µL of 1× SB was added to the glass vial and heated at 85 °C for 20 min. To avoid selection of sequences binding to the glass, the supernatant was transferred to an empty glass vial and incubated for 1 h at RT. Then, the supernatant was recovered, precipitated as described above, and the resulting pellet was resuspended in ultrapure water to be used as a template for the PCR amplification.

For a negative selection, the folded ssDNA pool was first incubated with a dried mixture of saturated and monounsaturated fatty acids at room temperature for 1 h. Subsequently, the supernatant was transferred into a vial containing dried polyunsaturated fatty acid and incubated under same conditions. The supernatant, containing the unbound ssDNA pool, was transferred to an empty glass vial for an additional incubation. The unbound ssDNA pool was then recovered, precipitated, and the resulting pellet was used as a template for the PCR reaction. The positive and negative selection steps were performed six and four times, respectively, as indicated in Table 1.

### 3.3. Sequencing and Bioinformatics Analysis

The original ssDNA library and the obtained selected ssDNA from the positive cycles (cycle 4, cycle 6, and cycle 10) and the negative cycles (cycle 5 and cycle 9) were analyzed by high-throughput sequencing on an Illumina system (Illumina, Little Chesterford, UK), as previously described [35]. Briefly, DNA libraries were amplified by PCR, using the adaptor and indexing sequences required for Illumina multiplex sequencing. The PCR products were purified on a 3% agarose gel using the Monarch^®^ Gel Extraction Kit (New England Biolabs, Ipswich, MA, USA), according to the manufacturer’s instructions. The samples were then mixed and loaded into a flow cell containing 10% PhiX. Sequencing and de-multiplexing were performed, according to Illumina’s instructions. Approximately 1,500,000 sequencing reads were analyzed for each library. All FASTQ files were processed through a series of custom scripts that were used sequentially to analyze the results. In short, the sequences corresponding to the variable region between the constant sequences were recovered. Only sequences possessing a random region of 30 to 36 nucleotides were recovered. Sequences containing at least one base with a quality score (Q) less than 30 were then removed before the remaining sequences were stored in a FASTA format. The frequency of each sequence in the library was calculated, and any sequences with a frequency <0.001% in all libraries were removed to decrease the time of analysis. The remaining sequences (5745 unique sequences, in our case) were then sequentially clustered in 5438 families using a Levenshtein distance of 6 (i.e., sequences with no more than 6 substitutions, insertions, or deletions). The frequency of each family was then calculated for each cycle. The multiple alignment of the 200 most abundant families was performed by MultAlin [36]. The secondary structure of the aptamer was predicted using the mFold Web Server.

### 3.4. Quantitative Real-Time PCR

The target specificity of Apt^PP^ was assessed by quantitative real-time PCR, as described previously [19]. The lowest *Ct* value indicates higher amounts of bound DNA (higher selectivity). Normalized *Ct* values were calculated as a ratio to the *Ct*-dolichol mixture. Each tested metabolite (1.8 nmoles) was placed in an amber glass vial, dried under nitrogen, and incubated with 12.5 nM of folded Apt^PP^ at room temperature for 1 h. The unbound Apt^PP^ was removed, and each vial was washed three times with 1× selection buffer. The bound Apt^PP^ was recovered by adding 200 µL of water and heating at 85 °C for 20 min. The denatured Apt^PP^ was recovered, precipitated overnight, and washed as, described in Section 3.2. The supernatant of the last wash was carefully discarded, without touching to the pellet. Then, the pellets were dried and resuspended in 20 µL of sterile water. All qRT-PCRs were carried out with PowerUp^TM^ SYBR^TM^ Green Master Mix (Thermo Fisher Scientific, Waltham, MA, USA). The PCR mixture contained 1 µL of the test sample, 500 nM of each primer, 5 µL of PowerUp^TM^ SYBR^TM^ Green Master Mix, and distilled water to a achieve a final volume of 10 μL. The samples were thermally cycled by real-time PCR as follows: 50 °C for 2 min, 95 °C for 2 min, followed by 40 cycles of 15 s each at 95 °C, 1 min at 55 °C, and 1 min at 72 °C. The negative controls, containing no template DNA, were included in each batch of qRT-PCR tests. The threshold cycle (*Ct*) values were set as the cycle at which the measured fluorescence intersected the cycle threshold line and were obtained using StepOnePlus^TM^ (Applied Biosystems, Waltham, MA, USA).

### 3.5. Non-Equilibrium Capillary Electrophoresis of Equilibrium Mixtures (NECEEM)

The efficacy of aptamer selection through utilizing lipids coated on a glass surface was confirmed using the method described by Berezovski et al., with a few modifications [19], in a PA 800 Plus Capillary Electrophoresis System (Beckman coulter^®^, Brea, CA, USA). Briefly, an uncoated fused silica capillary, with an internal diameter of 75 μm and an external diameter of 375 μm (eCAPTM Capillary Tubing, Beckman Coulter), a total length of 60 cm, and length to the detection window of 50 cm, was prepared and four rinsing steps were performed using 15 μL of 100 mM HCl, then 15 μL of ddH_2_O, followed by 15 μL of 100 mM NaOH, and finally rinsed with 15 μL of ddH_2_O. The steps using HCl or NaOH were performed for 30 min at 20 psi/137 kPa, while the rinsing steps using ddH_2_O were performed for 5 min 20 psi/137 kPa. After rinsing, the capillary was calibrated using 25 mM Tris Acetate pH 8.5 for 20 min at 20 psi/137 kPa.

In parallel, 10 nM of ssDNA pool (library), labeled with FAM, was folded, as described in Section 3.2. Two samples of the folded library were prepared. One sample was directly injected into the capillary for 13 s at 2 psi/13.7 kPa, and the sample was run for 30 min at 375 V cm^−1^ with Laser Induced Fluorescence (LIF) detection. The other folded library was incubated with 0.25 nmoles of dolichol mixture coated on a glass vial at room temperature for 1 h. The supernatant containing unbound ssDNA was injected into the capillary using the same condition as for the control library. The migration time for the samples was determined by the detection of one defined peak.

### 3.6. Plasmodium falciparum In Vitro Culture

Parasite 3D7 and NF54 strains were obtained from the MR4 Malaria Reagent Repository (ATCC, Manassas, VA, USA) as part of the BEI Resources Repository, NIAID, NIH. The parasites were maintained in O^+^ human erythrocytes (Interstate Blood Bank, Memphis, TN, USA) at 5% hematocrit in RPMI 1640 media supplemented with 2 g/L glucose, 2.3 g/L sodium bicarbonate, 5.94 g/L HEPES, 5 g/L Albumax I, 50 mg/L hypoxanthine, and 20 mg/L gentamicin. The parasites were held at 37 °C under reduced oxygen conditions (5% CO_2_, 5% O_2_, and 90% N_2_). Synchronous cultures in the ring stage (>98%) were obtained by applying two consecutives cycles of 5% sorbitol treatment. The gametocyte stages were obtained using the *P. falciparum* NF54 strain grown in human pooled serum (Interstate Blood Bank, TN, USA) to a final concentration of 10%, as described previously [16].

The generation of the *P. falciparum* strain with an inducible knockdown of PfPPRD, the morphological characterization, and metabolomics analysis were previously reported [16]. To assess potential changes in the Apt^PP^ localization or signal due to the reduced expression of PfPPRD, the *P. falciparum* parasites were cultured, with or without 0.5 μM anhydrotetracycline (aTc), for 8 days. Samples were obtained and processed, as described in Section 3.7, for colocalization studies with Apt^PP^, PfBiP, and nuclear staining.

The response of Apt^PP^ to the chemical modulation of isoprenoid biosynthesis was assessed in synchronous cultures at the ring stage (5 to 10% parasitemia and 4% hematocrit), treated with 1 μM MMV00813829 for 15 h, and then processed for microscopy studies.

### 3.7. Fluorescence Microscopy

The Apt^PP^ aptamers were commercially obtained with either 5′-6-FAM (6-FAM-Apt^PP^) or 5′-Cy5 (Cy5-Apt^PP^) modification (Integrated DNA Technologies, Coralville, IA, USA).

The aptamers were resuspended in ultrapure water. Immunofluorescence microscopy was performed, as described previously, with the modifications described herein [37]. Briefly, a 1 mL of infected red blood cells (iRBC), with a parasitemia level of 5% and a hematocrit of 4%, was centrifuged at 1600× *g* for 3 min. The resulting pellet was then washed with phosphate-buffered saline solution (PBS) and subsequently resuspended in 200 µL PBS, followed by the addition of 200 µL fixative solution (8% paraformaldehyde and 0.015% glutaraldehyde, EM grade, in PBS). Following a 30 min incubation at room temperature, the cells were centrifuged at 200× *g* for 3 min, and then gently washed and permeabilized by treatment with 500 µL of 0.1% Triton X-100 in PBS for 10 min, while gently rocking at room temperature. After permeabilization, the cells were treated with 500 µL of 0.1 mg/mL NaBH_4_ for 10 min at room temperature. The cells were blocked with 1 mL of 3% BSA for 1 h and incubated overnight with the primary antibody at 4 °C. Then, the cells were washed three times with PBS and resuspended in 250 µL of 3% BSA solution containing Alexa Fluor 594 secondary IgG antibodies (1:100 dilution, Life Technologies, Thermo Fisher Scientific, Waltham, MA, USA) and 0.25 µM of 6-FAM-Apt^PP^. Following incubation for 1 h at room temperature, the samples were washed three times with PBS, seeded for 1 h on coverslips pre-coated with poly-L-lysine, and then mounted on ProLong diamond with 4′,6′-diamidino-2-phenylindole (DAPI) (Invitrogen, Thermo Fisher Scientific, Waltham, MA, USA). The primary antibodies used for immunofluorescence microscopy in this study were the following: rat anti-PfBiP MRA-1247 (BEI Resources, NIAID, NIH, 1:100), rabbit anti-Cpn60 (1:1000), and rabbit anti-PfERD2 (1:2000), a gift from Dr. Vasant Muralidharan (University of Georgia).

The potential co-localization of Apt^PP^ and the mitochondria was assessed by MitoTracker^TM^ labeling (Invitrogen, Thermo Fisher Scientific, Waltham, MA, USA). Briefly, 100 µL of iRBC culture were spun down, washed one time with PBS, and resuspended in 100 µL PBS containing 50 nM MitoTracker^TM^. The parasites were incubated at 37 °C for 15 min and protected from light. The cells were washed three times with PBS, and a thin blood smear was performed, followed by fixation with methanol and blocking with 3% BSA, as described above. Then, 0.5 mL of 0.5 µM 6-FAM-Apt^PP^ was added on top of the thin blood smear, followed by incubation for 1 h. The slide was washed three times, air dried, and mounted on DAPI, as described above, and covered with a coverslip.

The parasites were also labeled with BODIPY 493/503 (Molecular Probes, Thermo Fisher Scientific, Waltham, MA, USA), as previously reported, with some modifications [19]. Briefly, 1 mL of iRBC culture was washed once with PBS, resuspended in 100 µL of PBS, and stained with 10 µM BODIPY for 15 min at 37 °C. The cells were washed three times with PBS and fixated with 8% paraformaldehyde and 0.015% glutaraldehyde, as described above. The cells were centrifuged at 200× *g* for 3 min, washed three times with PBS, and resuspended in PBS containing 3% BSA. The cells were washed once with PBS and incubated with 100 µL of 0.25 µM folded Cy5-Apt^PP^ for 1 h, followed by three washes, seeded on a treated coverslip, and mounted on DAPI, as described above.

Image processing, analysis, and display were preformed using a DeltaVision II microscope system with an Olympus IX-71 inverted microscope using a 100× objective. Image processing and analysis were performed using DeltaVision softWoRx software version 7.0.0 (GE Healthcare Life Sciences, Marlborough, MA, USA) and Adobe Photoshop 21.2.0. Colocalization analyses were performed using Fiji (Coloc 2) [38]. Adjustments to brightness and contrast were performed for display purposes.

## Figures and Tables

**Table 1:** Selection conditions used during each round of SELEX, as described in Figure 1. The incremental increase in washes was designed to promote the selection of high-affinity ligands, contributing to the iterative enrichment of the desired ligand sequences throughout the SELEX procedure.

Selection Round	ssDNA [nM]	Dolichol Mix (nmol)	Fatty Acid Mix (nmol)	Washing Steps
1	15	1.25	-	1
2	15	1.25	-	1
3	15	-	1.25	-
4	12.5	0.5	-	2
5	12.5	-	1.25	-
6	12.5	0.5	-	2
7	10	-	1.25	-
8	10	0.25	-	3
9	10	-	1.25	-
10	10	0.25	-	3

**Table 2:** Frequencies (in %) of the six most abundant SELEX families in different rounds. The percentages of each family in the library were analyzed after the positive (R04, R06, R09) or negative (R05, R10) selection rounds. The initial percentage of each family was also determined in the original library (R00). The constant regions (cst) are 5′-GCCTGTTGTGAGCCTCCT-3′ at the 5′-end, and 5′-GGGAGACAAGAATAAGCG-3′ at the 3′-end. The sequence similarities between families 1 and 2 are indicated in bold letters.

Family	Sequence (5′ → 3′)	R00	R04	R05	R06	R09	R10
1 (Apt^PP^)	cst-AT**GTCGA**CT**GG**CG**TTGGGAAA**TGCCGCTTAGTGG-cst	0.002	0.010	0.010	2.865	5.278	13.919
2	cst-**GTCGA**AA**GG**TT**TTGGGAAA**GCACCTCAGTTCTTGAG-cst	0	0.023	0.002	0.001	0.010	0.040
3	cst-AGATCGGAAGAGCACACGTCTGAACTCCAGT-cst	0	0.080	0.002	0	0.028	0.025
4	cst-GTCACGACGACGTCTCGTATGCCGTCTTCTGCTTG-cst	0	0.055	0	0	0.035	0.043
5	cst-ACTAACCATGCTCATCATGTGGTTCCGTATTAGG-cst	0	0	0	0	0	0.003
6	cst-GTGTCGTGTCTTGACTATCAAGCTAGTCTCTTTT-cst	0	0.002	0	0	0	0.005

**Table 3:** Primary sequences of Apt^PP^, Apt^PPInv^, and Apt^Scrambled^. The conserved motif (ATGTCGACTG) was identified using the MEME Suite program.

Aptamer	Sequence (5′ → 3′)
Apt^PP^	gcctgttgtgagcctcctATGTCGACTGGCGTTGGGAAATGCCGCTTAGTGGcgcttattcttgtctccc
Apt^PPInv^	cgcttattcttgtctcccATGTCGACTGGCGTTGGGAAATGCCGCTTAGTGGgcctgttgtgagcctcct
Apt^Scrambled^	cgcttattcttgtctcccTGGCTTCTAGAAATACCGTGGCGGTGTGAGGGTCgcctgttgtgagcctcct

## Data Availability

The data presented in this study are available in article and Supplementary Material.

## Supplementary Materials

The following supporting information can be downloaded at: https://www.mdpi.com/article/10.3390/molecules29010178/s1, Figure S1: Migration time of the folded ssDNA library at different concentrations; Figures S2–S4: Migration time of the folded ssDNA library at different concentrations; Table S1: Frequency of the different families; Table S2: Frequency of the different sequences of the families; Table S3: Multiple alignments of the 200 most abundant families.
