# Supplementary material for: Aptamer-Based Imaging of Polyisoprenoids in the Malaria Parasite
Source: Molecules. 2023 Dec 28;29(1):178. doi: 10.3390/molecules29010178 (PMC10780415; doi:10.3390/molecules29010178)
Supplement: Supplementary file 1 [file molecules-29-00178-s001.zip › molecules-2773587-Supplementary Materials.pdf]

Supplementary Materials

Aptamer-based Imaging of Polyisoprenoids in the Malaria Parasite

Flavia M. Zimbres [1,2], Emilio F. Merino [1,2], Grant J. Butschek [1,2], Joshua H. Butler [1,2], Frédéric Ducongé [3,4,5] and Maria B. Cassera [1,2,*]

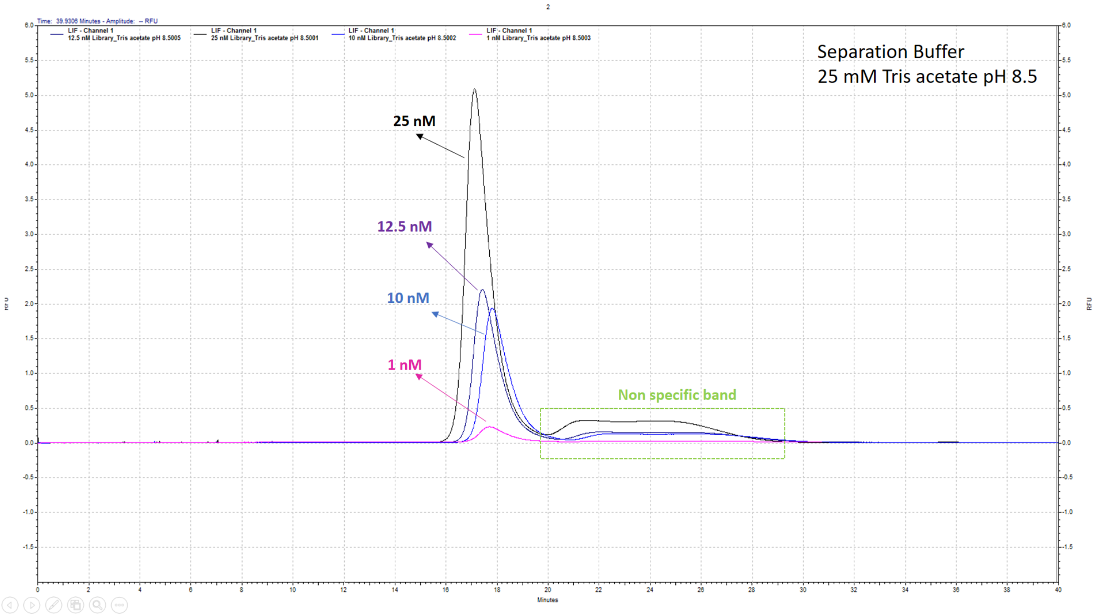

Figure S1. Migration time of the folded ssDNA library at different concentrations.

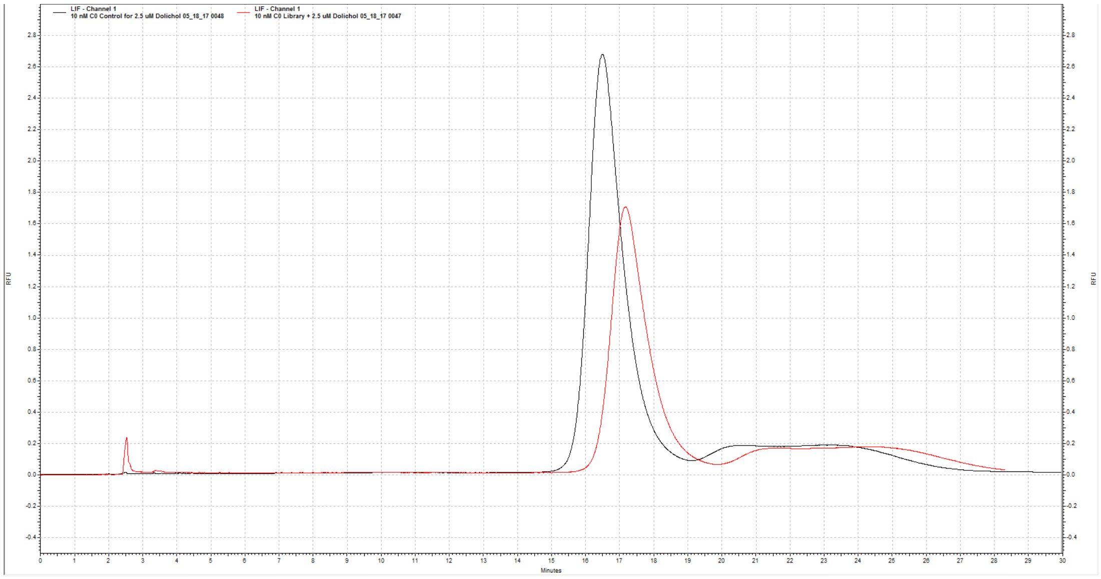

Figure S2. Migration time of the folded ssDNA library supernatant (10 nM) before (black line) and after incubation with 0.25 nmoles of the dolichol mixture (red line).

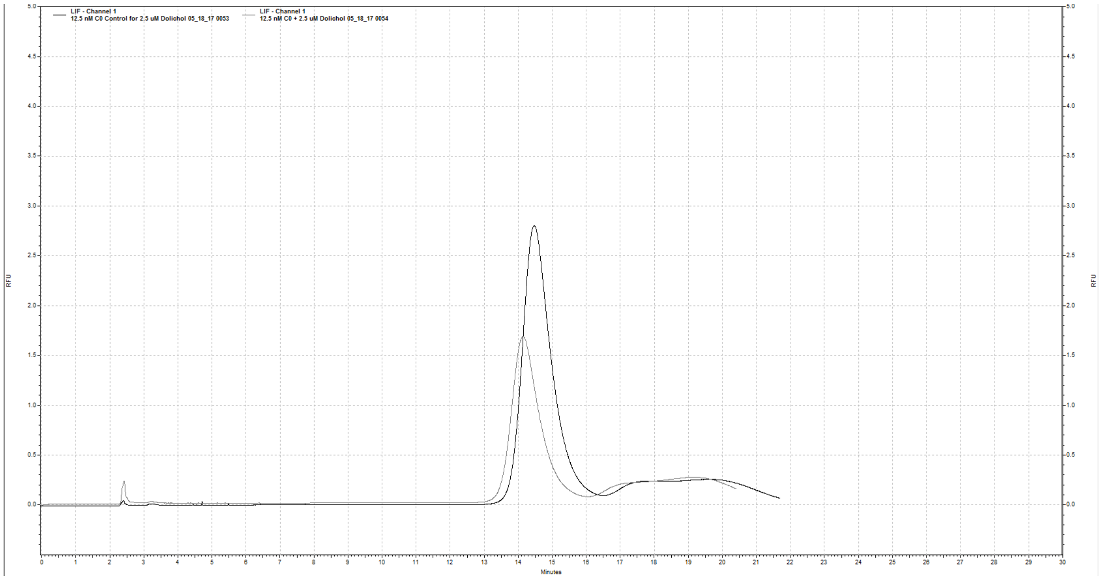

Figure S3. Migration time of the folded ssDNA library supernatant (12.5 nM) before (black line) and after incubation with 0.25 nmoles of the dolichol mixture (gray line).

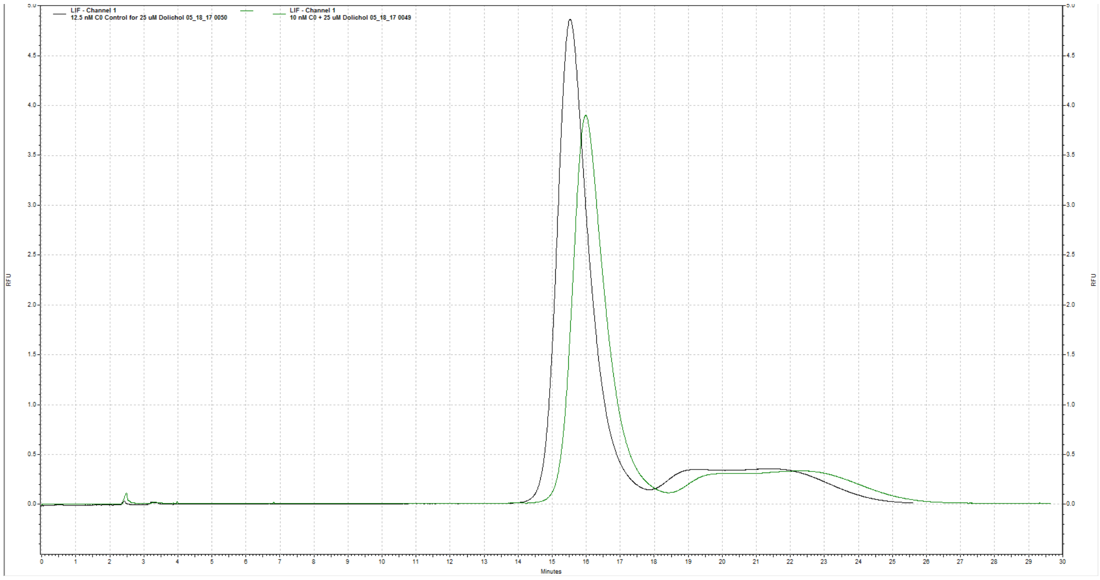

Figure S4. Migration time of the folded ssDNA library supernatant (12.5 nM) before (black line) and after incubation with 2.5 nmoles of the dolichol mixture (green line).
